# Late-Onset Intracranial Hemorrhage Presenting as Refractory Hyponatremia: A Case Report

**DOI:** 10.7759/cureus.38810

**Published:** 2023-05-10

**Authors:** Young Lee, Daisuke Son, Shintaro Imaoka, Tsubasa Nakai, Minako Kamimoto, Toshihiro Hamada, Shin-ichi Taniguchi, Masahiko Koda

**Affiliations:** 1 Department of Community-Based Family Medicine, Faculty of Medicine, Tottori University, Yonago, JPN; 2 Department of General Medicine, Hino Hospital, Hino-gun, JPN; 3 Tottori Medical Career Support Center, Tottori University Hospital, Yonago, JPN; 4 Department of Internal Medicine, Hino Hospital, Hino-gun, JPN

**Keywords:** head trauma, delayed intracranial hemorrhage, refractory hyponatremia, syndrome of inappropriate secretion of antidiuretic hormone, cerebral salt-wasting syndrome

## Abstract

Here, we report a case of refractory hyponatremia and delayed intracranial hemorrhage following a head injury. A 70-year-old male patient was admitted with complaints of left chest pain and light-headedness after a fall. Hyponatremia recurred despite the correction with intravenous saline. Head computed tomography revealed a chronic subdural hematoma. The subsequent introduction of tolvaptan improved hyponatremia and disorientation. Delayed intracranial hemorrhage is a differential cause of refractory hyponatremia after head contusion. This case is clinically relevant because (i) the diagnostic delay of late-onset intracranial hemorrhage is common but fatal, and (ii) refractory hyponatremia can be a hint of late-onset intracranial hemorrhage.

## Introduction

The causes of impaired consciousness are manifold. Cerebrovascular disorders, including subdural hematoma, can be recalled in the case of consciousness disorders after contusion, but we experienced a case of hyponatremia after head contusion, which resulted in consciousness disorders. In the present case, once the hyponatremia normalized after treatment with saline solution, the patient developed hyponatremia again along with neurological abnormalities. Therefore, a head computed tomography (CT) was performed, which revealed a delayed subdural hematoma. In refractory hyponatremia, delayed intracranial hemorrhage should be considered in the differential diagnosis. We report this case because the delay in the diagnosis of late-onset intracranial hemorrhage is common but fatal, and refractory hyponatremia may be a sign of late-onset intracranial hemorrhage.

## Case presentation

**Figure 1:**
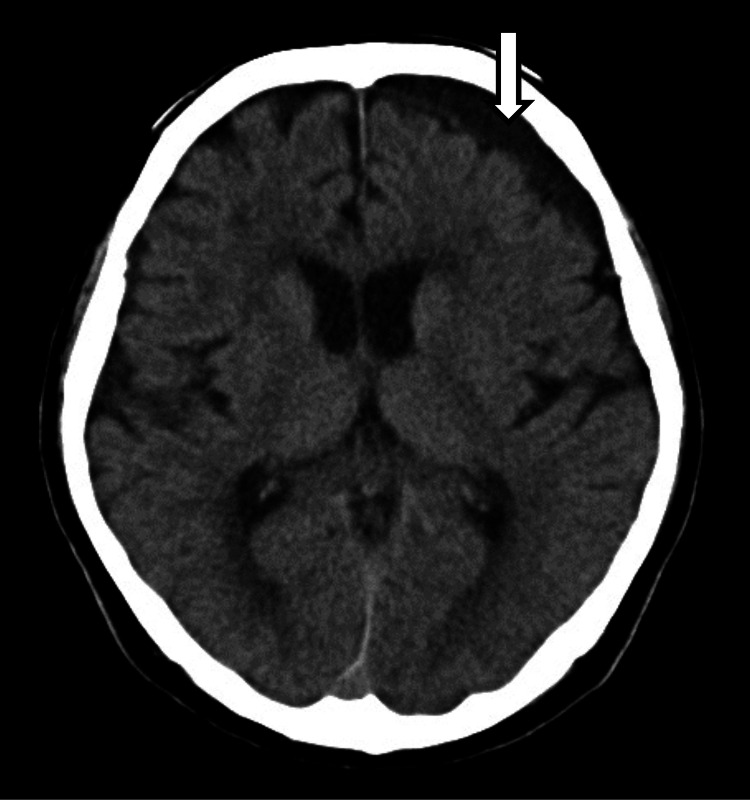
Head CT obtained 13 days after admission showing chronic subdural hematoma on the left (white arrow).

An independent 70-year-old male patient who consumed 600 mL of shochu (118 g of alcohol) per day was admitted with complaints of left chest pain and light-headedness after a fall. He had not been diagnosed with alcoholism or liver cirrhosis. On admission, his body temperature was 36.6°C, blood pressure was 132/100 mmHg, pulse was 72 beats per minute, and oxygen saturation was 94% in ambient air. A neurological examination was normal and did not reveal any abnormality. A CT scan of the chest revealed a fracture and hemothorax in the posterior arch of the left eighth to eleventh ribs and extensive shadows suggestive of a pulmonary contusion in the right lung. A CT scan of the head showed no abnormality. The blood sample revealed a low serum sodium level of 108 mEq/L, and the patient was referred to the Department of General Medicine. About one month before admission, his serum sodium was 143 mEq/L. Laboratory tests revealed a hemoglobin count of 13.6 mg/dL (reference range: 13.5-17.5 mg/dL), blood urea nitrogen count of 16.8 mg/dL (reference range: 7­-20 mg/dl), and creatinine count of 1.09 mg/dL (reference range: 0.9-1.3 mg/dl). Thyroid hormones and cortisol were normal. He had no ascites or lower extremity edema. There was no evidence of liver cirrhosis on the CT scan and he did not have hepatic encephalopathy. The hypo-osmolality (233 mOsm/kg) and normal extracellular fluid (UNa of 47 mEq/L, UOsm of 537 mOsm/kg H_2_O) suggested syndrome of inappropriate secretion of antidiuretic hormone (SIADH). Antidiuretic hormone (ADH) level was 0.8 pg/mL on day X+9 which was within reference limits, and the lack of suppression despite low serum sodium also suggested SIADH. The serum sodium level normalized on day X+5 and the intravenous infusion was terminated on the same day. Serum sodium levels normalized, but cognitive decline and light-headedness were observed. Rehabilitation was continued, considering the possibility of delirium and decline in activities of daily living (ADLs) associated with hospitalization, but a blood sample taken again on day X+9 revealed a low serum sodium level of 118 mEq/L. Blood was drawn on day X+13, considering the patient’s progress with water restriction, revealing a low serum sodium level of 105 mEq/L. On the same day, a head CT scan was performed to investigate the head lesion due to poor right upper and lower limb movements and cognitive decline, which revealed a left chronic subdural hematoma (Figure 1). The neurosurgeon provided medical information stating that surgical treatment was not indicated at this time.

**Figure 2:**
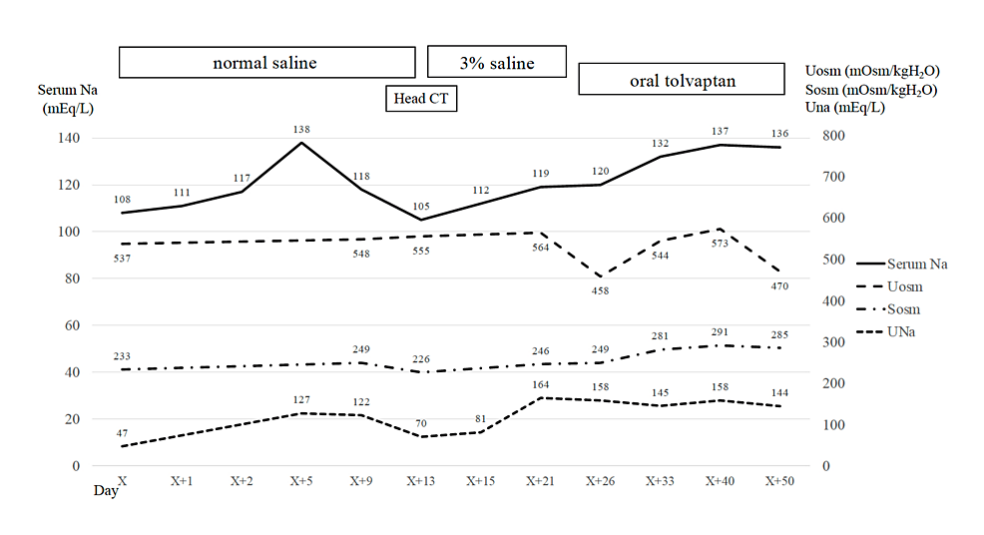
Patient history and laboratory values.

Despite the administration of normal saline, the patient’s serum sodium level decreased; hence, the patient began receiving a 3% saline infusion from day X+13. An oral tolvaptan dose of 7.5 mg was started on day X+26, and his serum sodium level normalized to 137 mEq/L two weeks later. Oral tolvaptan was tapered to 1.875 mg, but the patient’s serum sodium level was maintained, allowing him to be discharged from the hospital on day X+62. The patient history and laboratory values are shown in Figure 2. At the outpatient clinic after discharge, he was able to discontinue tolvaptan, and the hyponatremia has not recurred.

## Discussion

The differentiation of consciousness disorders varies widely, and determining the cause of the disorder and understanding the pathophysiology in a short period is necessary for the emergency setting. A traumatic cerebrovascular disorder is the first differential in the case of consciousness disorder after a head contusion. However, hyponatremia should be considered in the differential of delayed disturbance of consciousness after trauma. The delay in diagnosis of late-onset intracranial hemorrhage can be fatal, and refractory hyponatremia can be a sign of late-onset intracranial hemorrhage.

On admission, the patient was unsteady but not disoriented. We judged the patient to have an SIADH pattern based on hyponatremia, normal extracellular fluid volume, low serum osmolality, UNa of 47 mEq/L, and UOsm of 537 mOsm/kg H_2_O. We suspected symptomatic hyponatremia because of the patient’s light-headedness and corrected it by intravenous saline injection. However, the patient developed a delayed loss of consciousness, and a close examination revealed a hyponatremia exacerbation and chronic subdural hematoma. The patient was given a follow-up observation for the chronic subdural hematoma and water restriction was performed for the hyponatremia based on the suspicion of SIADH.

The most common form of delayed hyponatremia after head contusion is cerebral salt-wasting syndrome (CSWS), which is a form of hyponatremia that occurs within one week to 10 days after subarachnoid hemorrhage, head trauma, intracranial neoplasms, infectious or cancerous meningitis, encephalitis, and intracranial surgery [1]. It is caused by increased natriuretic peptide secretion, decreased sodium, uric acid, and water reabsorption from the proximal tubules due to decreased sympathetic stimulation of the paraglomerular apparatus, decreased renin-aldosterone secretion, resulting in markedly increased water and salt excretion, and decreased extracellular fluid.

Distinguishing CSWS from SIADH is difficult in clinical practice because both conditions cause an increase in urine osmolality (due to decreased circulating plasma volume in CSWS and inappropriate ADH secretion in SIADH), urinary sodium of >40 mEq/L (due to inappropriate urinary sodium excretion in CSWS and increased circulating plasma volume in SIADH), decreased serum uric acid levels (due to increased uric acid excretion in the proximal tubules, endocrine factors such as brain natriuretic peptide, sympathetic nervous system failure in CSWS, increased circulating plasma volume, and direct effect of ADH on V1 receptors in SIADH), other nonsignificantly different biochemical indices, and association with central nervous system diseases [2,3]. This is considered important for determining its treatment strategy. If we assume that this case is SIADH, SIADH of thoracic origin was also on the differential because a hemothorax was also present on admission. Because hyponatremia progressed again after treatment and its course was paralleled by findings of cognitive decline and subdural hematoma, a central origin of SIADH is likely.

The key factors in differentiating CSWS from SIADH are fluid volume, serum brain natriuretic peptide level, and serum sodium-corrected uric acid levels. The appearance of impaired consciousness after water restriction and exacerbation of hyponatremia in the present case suggests CSWS, but the brain natriuretic peptide level is difficult to evaluate because tolvaptan was started midway through the course of the patient’s illness. The serum sodium-corrected uric acid level improved after tolvaptan administration, which is a common finding in SIADH. Thus, completely differentiating CSWS from SIADH was difficult in this case.

It has been reported that traumatic brain injury can cause hyponatremia, mostly due to dysregulation of the neuroendocrine system [4-7]. Although most cases of hyponatremia after traumatic brain injury are transient and reversible, some cases, such as the present case, may have a persistent or recurrent course, which is key to suspecting delayed intracranial hemorrhage. Because intracranial hemorrhage can be fatal if detected late, it is important for physicians to suspect cerebral abnormality, including subdural hematoma, when encountering refractory hyponatremia after head trauma, even if the head CT just after the trauma reveals no findings.

## Conclusions

We experienced a case of refractory hyponatremia as a cause of delayed altered mental status after a head contusion. Physicians should suspect cerebral abnormality, including late-onset intracranial hemorrhage, when seeing refractory hyponatremia after trauma, even if the initial imaging reveals no findings.

## References

[1] Leonard J, Garrett RE, Salottolo K, Slone DS, Mains CW, Carrick MM, Bar-Or D (2015). Cerebral salt wasting after traumatic brain injury: a review of the literature. Scand J Trauma Resusc Emerg Med.

[2] Palmer BF (2003). Hyponatremia in patients with central nervous system disease: SIADH versus CSW. Trends Endocrinol Metab.

[3] Sterns RH, Silver SM (2008). Cerebral salt wasting versus SIADH: what difference?. J Am Soc Nephrol.

[4] Donati-Genet PC, Dubuis JM, Girardin E, Rimensberger PC (2001). Acute symptomatic hyponatremia and cerebral salt wasting after head injury: an important clinical entity. J Pediatr Surg.

[5] Chang CH, Liao JJ, Chuang CH, Lee CT (2008). Recurrent hyponatremia after traumatic brain injury. Am J Med Sci.

[6] Shen B, Li L, Li T (2017). Concurrence of inappropriate antidiuretic hormone secretion and cerebral salt wasting syndromes after traumatic brain injury. Front Neurosci.

[7] Chua TH, Ly M, Thillainadesan S, Wynne K (2018). From renal salt wasting to SIADH. BMJ Case Rep.

